# High expression of the long non-coding RNA HEIRCC promotes Renal Cell Carcinoma metastasis by inducing epithelial-mesenchymal transition

**DOI:** 10.18632/oncotarget.14149

**Published:** 2016-12-24

**Authors:** Jing Xiong, Ying Liu, Shengjun Luo, Li Jiang, Yang Zeng, Zhixiong Chen, Xiaobo Shi, Bufan Lv, Wei Tang

**Affiliations:** ^1^ Department of Urology, The First Affiliated Hospital of Chongqing Medical University, Chongqing; ^2^ Departmentof Urology and Andrology, Taihe Hospital, Hubei University of Medicine, Shiyan, Hubei Province, China; ^3^ Department of Preventive Medicine, School of Public Health and Management, Hubei University of Medicine, Shiyan, Hubei Province, China

**Keywords:** long non-coding RNAs, HEIRCC, renal cell carcinoma, prognosis, EMT

## Abstract

Increasing evidence indicates that long non-coding RNAs (lncRNAs) have been associated with cancer development. However, the contributions of lncRNAs to renal cell carcinoma (RCC) remain poorly characterized. Here, we identified a novel lncRNA, termed HEIRCC, which was up-regulated in RCC tissues through lncRNA microarray analysis and subsequent validation in 60 RCC clinical specimens and cell lines. The high expression of HEIRCC is associated closely with the clinical pathology features such as larger tumor size, poor differentiation, lymphatic metastasis. *In vitro* assays revealed that HEIRCC knockdown could inhibit cell proliferation, trigger late apoptosis, suppress cell migration and invasion. We further demonstrated that depletion of HEIRCC reduce the epithelial to mesenchymal transition (EMT) program by regulating expression levels of EMT-associated markers in RCC cells. Thus, HEIRCC might be act as an important regulator of EMT in RCC progression and might be a novel therapeutic target for the advanced RCC therapy.

## INTRODUCTION

Renal cell carcinoma (RCC) counts among the major cancers that endanger human health in the world, with about 63,920 newly detected cases yearly [1]. Renal cell carcinoma incidence has increased for over two decades, and patients with advanced RCC (stage IV) have a significantly reduced five-year survival rate (less than 30%) [2, 3]. Despite the identification of several genes related to cancer development and progression malignant events still need to be characterized at the molecular level. Indeed, uncovering the exact molecular mechanisms by which renal cell carcinoma develops and progresses is essential, in order to identify novel therapeutic targets to combat RCC. Epithelial–mesenchymal transition (EMT) was initially reported for its crucial role in metazoan embryogenesis as well as organ development. Emerging evidence has shown that cancer progression and metastasis are tightly associated with EMT [4, 5].

Long non-coding RNAs (lncRNAs), a subclass of noncoding RNAs (ncRNAs), have sequence lengths of 200 bp and above [6, 7]. At the beginning, lncRNA molecules were merely described as cloning artifacts. However, it has become increasingly apparent that lncRNAs are involved in multiple cellular biological processes, ranging from transcriptional and post-transcriptional regulation to cell cycle distribution, cell differentiation and epigenetic modifications [8]. Interestingly, lncRNAs attract increasing attention for the elucidation of interconnected pathways regulating tumorigenesis and metastasis in multiple malignancies, including glioma, lung, colorectal and hepatocellular cancers [9, 10]. Meanwhile, reports assessing lncRNA involvement in RCC are scarce, and the pathways regulating RCC aggressiveness remain poorly understood.

This study aimed to assess lncRNA involvement in the control of EMT as well as metastasis in RCC. The differences between lncRNA expression profiles of RCC and tumor-adjacent non-tumor tissues were assessed by lncRNA microarrays. The up-regulated lncRNAs were validated for their expression levels in another cohort of patients. In addition, we characterized the pathologic relevance of the lncRNA TCONS_00006756, also termed HEIRCC (high-expressed in renal cell carcinoma), in RCC development and progression. Furthermore, mechanistic investigations into the function of HEIRCC in RCC were performed through loss-of-function studies, which provided new insights into the role of lncRNAs in RCC development and progression. The current findings suggest a potential application for HEIRCC in the treatment of RCC.

## RESULTS

### HEIRCC is strongly up-regulated in the RCC tumor tissues and cancer cells

**Figure 1:**
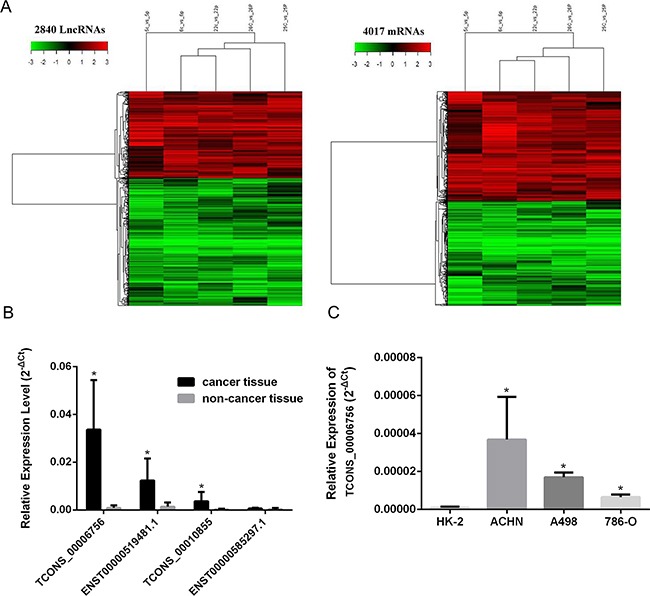
HEIRCC expression in RCC cell lines and tissues **A**. Hierarchical clustering analysis of lncRNAs and mRNAs that were differentially expressed ( > *2*-fold; *P* < *0.05*) in 5 pairs of RCC and nontumor samples (c, cancer tissues; p, paired nontumor samples; 5-6-22-25-26, patient number). Red represents high expression and green represents low expression. **B**. The expression of four selected lncRNAs including HEIRCC in RCC patients. **C**. The relative expression of HEIRCC mRNA in renal cancer and normal cell line.*P < 0.05.

Hierarchical clustering showed 2,840 (10%) lncRNAs that were differentially expressed (cutoff fold change value, 2; *P<*0.05) between cancer samples and non-tumor specimens. These comprised 1,151 up-regulated and 1,688 down-regulated. Further, a total of 2,056 (7.4%) protein-coding mRNAs were consistently increased; meanwhile, 1,961 (7.05%) mRNAs showed decreased amounts (Figure 1A). These microarray results are available in NCBI Gene Expression Omnibus (accession number: GSE88811). The 100 lncRNAs and protein-coding genes showing the highest fold changes are summarized in Supplementary Table 1. Criteria with higher stringency were used to obtain lncRNAs for subsequent assessments: (1) elevated or reduced amounts; and (2) similar expression patterns in various specimens; (3) a fold-change in expression of >10. Interestingly, 4 and 7 lncRNAs were markedly downregulated and upregulated in RCC tissues, respectively. We focused mainly on the upregulated lncRNAs because they can be used more readily than down-regulated lncRNAs as early diagnosis markers or therapeutic targets. Some of them were further validated in an additional cohort of 55 samples by real-time PCR (*P<0.05; Figure 1B). A non-annotated transcript, TCONS_00006756, was identified as one of the most significantly up-regulated entities in RCC tumor tissues in comparison with corresponding adjacent tissue specimens. As shown in Figure 1C, the expression levels of TCONS_00006756 were starkly elevated in RCC cells compared with HK-2 amounts (*P<0.05). These findings suggested that TCONS_00006756 might act as an oncogenic lncRNA in RCC carcinogenesis; the lncRNA is hereafter referred to as HEIRCC. Information from the GenBank revealed that HEIRCC is located on the reverse strand of human chromosome 3:194021490-194023848, and has a 340 bp long transcript.

### HEIRCC expression is correlated with RCC progression

To determine whether HEIRCC expression levels are related to RCC progression, we analyzed the association of HEIRCC with clinicopathological factors in RCC patients. Statistical analyses revealed that high expression of HEIRCC in RCC patients was significantly correlated with tumor stage, histological grade, lymph node metastasis, and distant metastasis, as shown in Table 1 (*P*<0.05). Therefore, overexpression of HEIRCC is involved in RCC malignancy.

**Table 1:** Correlation between HEIRCC expression and clinicopathologic factors in RCC patients (n=60)

Clinicopathological parameters	Case no.	HEIRCC expression		*X^2^*	*P*
		**High no.(%)**	**Low no.(%)**		
Age(years)				1.609	0.205
≤60	34	14(41.2%)	20(58.8%)		
>60	26	15(57.7%)	11(42.3%)		
Gender				0.601	0.438
Male	29	13(44.8%)	16(55.2%)		
Female	31	17(54.8%)	14(45.2%)		
Histological grade				4.356	0.037
Well	45	19(42.2%)	26(57.8%)		
Moderate & Poor	15	11(73.3%)	4(26.7%)		
Tumor stage				3.937	0.047
I & II	47	18(38.3%)	29(61.7%)		
III & IV	13	9(69.2%)	4(30.8%)		
Lymph node metastasis				4.375	0.036
Positive	10	7(70.0%)	3(30.0%)		
Negative	50	21(35.0%)	39(65.0%)		
Distant metastasis				4.271	0.039
Positive	12	9(75.0%)	3(25.0%)		
Negative	62	20(41.7%)	28(58.3%)		

### HEIRCC affects cell proliferation and apoptosis

**Figure 2:**
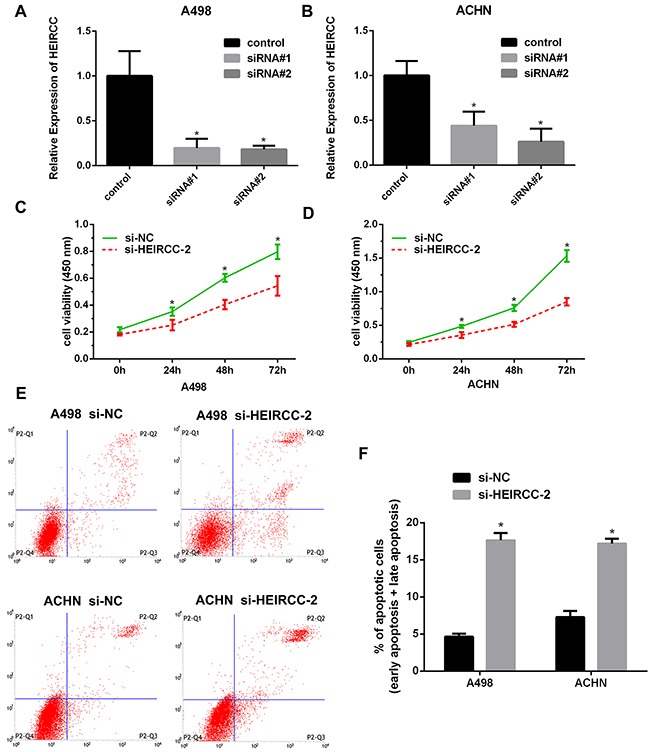
Effects of HEIRCC on RCC cell proliferation and apoptosis **A, B**. Decreased expression of HEIRCC was detected after transfection of HEIRCC-siRNA and siRNA control. **C, D**. HEIRCC depletion inhibited the proliferation of A498 and ACHN. **E, F**. HEIRCC knockdown promoted RCC cells apoptosis.*P < 0.05.

To further assess the biological effect of HEIRCC in RCC malignancy, loss-of-function studies were performed. HEIRCC expression levels were efficiently reduced in cancer cells transfected with HEIRCC-siRNA#2. Therefore, HEIRCC-siRNA#2 was used in subsequent functional experiments (*P<0.05, Figure 2A, 2B). Interestingly, *in vitro* CCK-8 assays demonstrated that HEIRCC knockdown dramatically reduced the proliferative capacity of RCC cells in comparison with the siRNA scramble control (*P < 0.05, Figure 2C, 2D). Next, we analyzed cell apoptosis by flow cytometry. Increased apoptosis was observed in RCC cells transfected with HEIRCC-siRNA#2 (*P<0.05, Figure 2E, 2F). Collectively, these findings suggested that HEIRCC may play a pivotal role in the proliferation and apoptosis of RCC cells *in vitro*.

### HEIRCC is involved in RCC cell migration and invasion

**Figure 3:**
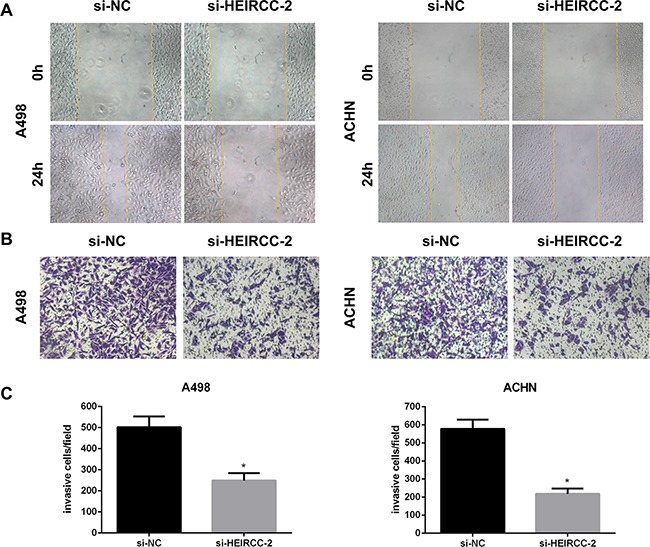
Effects of HEIRCC on RCC cell progression **A**. The migration and **B,C** invasion capacity of A498 and ACHN cells was decreased by HEIRCC-siRNA.*P < 0.05.

Cell migration and invasion are important prerequisites for tumor progression and metastasis. To assess whether HEIRCC modulates cell migration and invasion, wound healing test and matrigel invasion assay were performed. Our results demonstrated that RCC cell malignancy features were significantly suppressed after HEIRCC silencing by HEIRCC-siRNA#2 (*P<0.05, Figure 3A, 3B, 3C).

### HEIRCC regulates RCC cell progression via the EMT mechanism

**Figure 4:**
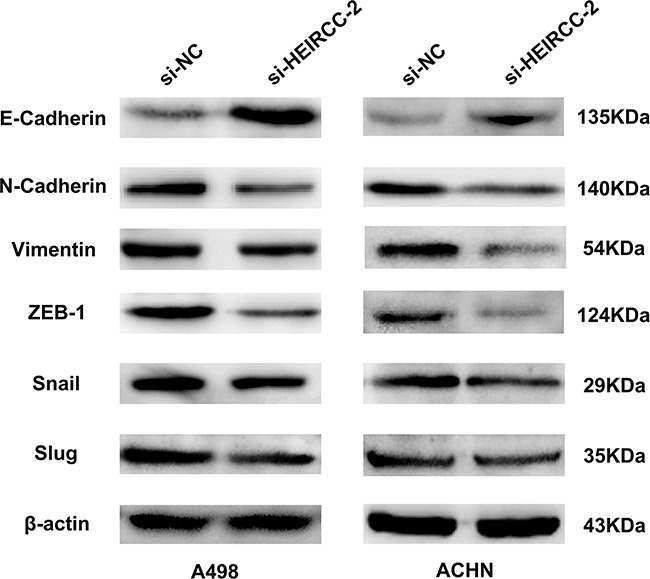
The expression of EMT related proteins were measured by western blot analyses after transfection

Since EMT is crucial for tumor propagation and metastasis, the effects of HEIRCC on EMT were analyzed. Interestingly, depletion of HEIRCC resulted in upregulated E-cadherin as well as downregulated N-cadherin and Vimentin. In addition, EMT transcription factors were analyzed, and partial loss of endogenous HEIRCC expression overtly decreased ZEB1 amounts, whereas Snail and Slug amounts showed no remarkable changes. Overall, the above findings pointed to HEIRCC as a new positive regulator of EMT, with a function in RCC malignancy (Figure 4).

## DISCUSSION

Despite the great therapeutic advances made in RCC, including surgical resection and adjuvant therapy, the long-term prognosis of RCC patients with distant metastases remains unfavorable. Increasing functional studies have indicated that lncRNAs, which may act as oncogenes or tumor suppressors, are involved in the carcinogenesis and progression of several tumors, including RCC [11, 12]. Although dysregulation of lncRNAs associated with RCC has been demonstrated, the functions and clinical significance of most dysregulated lncRNAs in the progression and aggressiveness of RCC remain unknown [13, 14].

In the current study, we identified the novel oncogenic lncRNA TCONS_00006756, termed HEIRCC, as one of the most up-regulated lncRNAs in RCC tissues compared with paired peritumoral tissues by lncRNA microarray analysis. This is consistent with previous studies by Blondeau et al. and Yu et al. [15, 16]. Moreover, further validation was performed in an additional cohort of RCC clinical tissue specimens and cell lines. High expression of HEIRCC is closely associated with large tumors, poor differentiation, and lymph node metastasis, and potentially correlated with RCC formation and progression.

Moreover, loss-of-function studies suggested HEIRCC silencing results in reduced cell growth, enhanced apoptosis, and inhibited aggressiveness. The above findings indicated HEIRCC might be a tumor oncogene in RCC.

EMT is crucial in normal organ development and human pathology, e.g. tumor aggressiveness. It is generally accepted that EMT is critical for tumor invasion as well as metastasis [17, 18, 19]. However, the underlying molecular mechanisms of EMT mediating the metastatic cascade remain largely unclear. Accumulating evidence indicates that lncRNAs can drive metastasis by inducing migration and invasion of cell through regulation of critical proteins [20]. In this study, we surprisingly observed that after HEIRCC knockdown, some hallmarks of mesenchymal cells such as N-cadherin and Vimentin showed reduced levels whereas E-cadherin (epithelial protein) amounts were increased. It has been reported that several transcription factors, including Snail, Slug and ZEB1 can initiate EMT via repression of E-cadherin expression by targeting the E-boxes in its promoter [21, 22]. We thus assessed whether HEIRCC regulates these transcription factors, and promotes tumor progression and metastasis in RCC. Western blotting analysis showed that knockdown of HEIRCC markedly repressed ZEB1 expression levels, while the other transcription factors such as Snail and Slug were not significantly changed. Overall, these findings indicated that HEIRCC promotes RCC progression by activating the EMT process in a ZEB1-dependent manner.

In summary, we have described, for the first time, the clinicopathologic features as well as the critical function of HEIRCC in RCC cell migration, invasion, and EMT. Our findings suggest that HEIRCC may serve as a novel therapeutic target for the advanced RCC treatment. Owing to the limited information obtained with a single siRNA, more specific siRNAs with high inhibition efficiency and a gain-of-function HEIRCC model system will be applied to comprehensively evaluate its biological function in subsequent studies. Moreover, the correlation between HEIRCC and the 5-year survival rate needs to be established with more patients.

## MATERIALS AND METHODS

### Patients and specimens

5 paired samples were prepared for lncRNA microarray analysis and 60 paired samples were used for an extra evaluation by real-time PCR. All patients were informed and have declared written informed consent that their samples can be used for research. This study was approved by the Research Ethics Committee of Chongqing Medical University, China. Patients received retroperitoneal laparoscopic radical nephrectomy, retroperitoneal laparoscopic partial nephrectomy, cytoreductiv enephrectomy at The First Affiliated Hospital of Chongqing Medical University and were diagnosed with ccRCC histopathologically after surgery. The tumor specimens were staged primarily according to UICC 2009 TNM tumor staging system [23], and histological graded based on the Fuhrman criteria and Union Internationale Contre le Cancer [24, 25]. All the samples were rapidly and carefully frozen in liquid nitrogen after resection until use. None of the patients had been pretreated with radiotherapy or chemotherapy prior to surgery.

### Cell culture

786-O, A498, ACHN RCC cell lines and immortalized normal human proximal tubule epithelial cell lines HK-2 were obtained from the Cell Bank of Type Culture Collection (Chinese Academy of Sciences, Shanghai, China). Cells were maintained at 37°C in a humidified incubator containing 5% CO_2_ in RPMI 1640 medium supplemented with 10% fetal bovine serum and antibiotics.

### Microarray and computational analysis

The microarray work was performed by CapitalBio Corporation, Beijing, China. Briefly, paired samples were used to synthesize the double-stranded complementary DNA (cDNA) by reverse-transcription polymerase chain reaction. The labeled cDNAs were hybridized to the Agilent Human LncRNA + messenger RNA (mRNA) Array V4.0 (4 × 180K, format). More than 40,916 lncRNAs were collected from authoritative data sources, including the GENCODE/Ensembl, Human LncRNA Catalog, RefSeq, USCS, ncRNA Expression Database (NRED), H-InvDB, LncRNAs-a (enhancer-like), RNAdb, CombinedLit, Antisense ncRNA pipeline, HoxncRNAs, UCRs, and Chen Runsheng lab. After hybridization and washing, the processed slides were scanned with the Agilent Microarray Scanner (Agilent technologies Santa Clara, CA, USA). Feature Extraction software 11.0.1.1 (Agilent Technologies) was used to analyze array images. Raw data were normalized by a Quantile algorithm using GeneSpring GX v11.5.1 software package (Agilent Technologies). A fold change of ≥ 2.0 and P ≤ 0.05 was set as the threshold for statistically significant up- and down-regulated genes, respectively. The FDR were calculated using Benjamini Hochberg method throughout the computational analysis. Hierarchical Clustering was performed based on differentially-expressed mRNAs and lncRNAs using Cluster_Treeview software from Stanford University.

### Real-time quantitative reverse transcription-PCR

Total RNA from 786-O, A498, ACHN and HK-2, as well as the RCC samples, and corresponding adjacent noncancerous kidney samples was isolated by RNAiso plus (TaKaRa, Dalian, China). Then RNA was reversely transcribed using a PrimeScript™ RT reagent Kits with gDNA Eraser (TaKaRa, Dalian, China). Real-time PCR was performed using the SYBR Green PCR Kit purchased from TaKaRa Biotechnology. The primers were designed and synthesized by Genepharma Co., Ltd. (Shanghai, China). GAPDH (forward 5’-GTCATCAATGGAAATCCCATCA-3’; reverse 5’-CCAG TGGACTCCACGACGTAC-3’ Size: 98bp); ENST 00000519481.1 (forward 5’-TCTGAGCCTGATGGATTTACAGTGA-3’; reverse 5’-TGTCATTCCAGTG CATGGTTCC-3’ Size:76bp); TCONS_00006756 (forward 5’-GCTGCTATTCTGGTGCCC-3’; reverse 5’-TCAACT CCGATAAACAGGTGA-3’ Size:159bp); TCONS_0001 0855 (forward 5’-AGAGGGTAGGGGATGGAAGTGG-3’; reverse 5’-AGGGATAGCAAACACCTAAAATGCA-3’ Size:143bp); ENST00000585297.1 (forward 5’-AGTTGGAA GGACTCCCCAGG-3’; reverse 5’-TAACCCACAAATG AAGTGCTACTCT-3’ Size:143bp).

### RNA interference and cell transfection

Specific siRNAs targeting TCONS_00006756 and a scrambled negative control (si-NC) were synthesized by Genepharma Co., Ltd. (Shanghai, China). A498 and ACHN were seeded onto the plate and reached 40% confluence on the second day, the siRNAs were transfected into the cells using Lipofectamine 2000 reagents (Life Technologies) according to the manufactuer's instruction. The efficiencies of RNA interference were determined by qRT-PCR after 48h transfection. TCONS_00006756-siRNA#1: GGAAGACUGUCACCCUCUUTT; TCONS_00006756-siRNA#2: UCACCUGUUUAUCGGAGUUTT.

### Cell proliferation assay

Appropriate cells that had been transfected with TCONS_00006756 siRNAs and negative control (NC) were seeded into 96-well plates in triplicate at densities of approximately 2000 cells per well. CCK-8 (Wanleibio, China) assay was used to analyze the cell viability at the indicated times.

### Flow cytometry analysis for apoptosis

After 48 h transfection as earlier described, the cells were collected and washed as well as resuspended with PBS. Then, cells were stained with FITC-Annexin V and PI (Beyotime Biotechnology, China) for apoptosis analysis with a FACScan flow cytometer (BD Bioscience). The experiments were performed in triplicates.

### Wound scratch assay

Cells from each group were seeded into 24-well plates at a density that reached 90-95% confluence as a monolayer. The monolayer was gently scratched across the center of the well with a 200μl plastic tip. After scratching, images were obtained at 0 and 24 h by using a microscope.

### Transwell invasion assay

Matrigel (BD Biosciences, USA) was thawed at -4°C overnight and diluted with serum-free RPMI-1640 medium (dilution, 1:8). A 60-μl sample of this mixture was deposited evenly into a 24-well chamber (8 μm, Millipore, USA), which was incubated for 30 min at 37°C. A total of 5×10^4^ cells were resuspended in 200μl of serum-free culture medium and loaded into the upper chamber (Millipore) whereas the bottom chamber was filled with 500 μl culture medium containing 10% FBS. The setup was incubated in the 37°C humidified CO_2_ for 48 h. Cells that passed through the filter were fixed with 4% paraformaldehyde and stained with 0.5% crystal violet. The numbers of invaded cells were counted in five randomly selected high power fields under a microscope (Olympus).

### Western blot and antibodies

Total proteins were lysed in RIPA buffer (Beyotime Biotechnology) supplemented with protease inhibitors. The protein concentrations were determined using BCA Protein Assay Kit (Beyotime Biotechnology). Identical quantities of proteins were separated on 10% SDS-PAGE and transferred to a PVDF membrane. After incubation with antibodies specific for Vimentin, N-Cadherin, and E-Cadherin, Slug, ZEB1, Snail and β-actin (Wanleibio, China). The bands were then scanned by a Imaging System and quantified by Quantity One v4.6.2 software (BIO-RAD).

### Statistical analysis

All the statistical analyses were performed using SPSS 19.0 software (SPSS, Chicago, IL, USA). All data were summarized and expressed as the mean ± SD. The differences between independent groups were analyzed using Student's *t*-test. Pearson's chi-square tests were used to analyse the correlation between lncRNA levels and clinical features. A *P* value less than 0.05 was considered to be statistically significant.

## SUPPLEMENTARY MATERIALS FIGURES AND TABLES




