# Supplementary material for: High expression of the long non-coding RNA HEIRCC promotes Renal Cell Carcinoma metastasis by inducing epithelial-mesenchymal transition
Source: Oncotarget. 2016 Dec 24;8(4):6555–63. doi: 10.18632/oncotarget.14149 (PMC5351652; doi:10.18632/oncotarget.14149)
Supplement: Supplementary file 1 [file oncotarget-08-6555-s001.pdf]

High expression of the long non-coding RNA HEIRCC promotes Renal Cell Carcinoma metastasis by inducing epithelial-mesenchymal transition

SUPPLEMENTARY TABLE

Supplementary Table 1: The top 100 dysregulated lncRNAs and mRNAs identified by microarrays in 5 pairs of RCC tissues.

See Supplementary File 1
